# How DNA methylation affects the Warburg effect

**DOI:** 10.7150/ijbs.45420

**Published:** 2020-04-27

**Authors:** Xingxin Zhu, Zefeng Xuan, Jun Chen, Zequn Li, Shusen Zheng, Penghong Song

**Affiliations:** 1 Division of Hepatobiliary and Pancreatic Surgery, Department of Surgery, First Affiliated Hospital, School of Medicine, Zhejiang University; 2 NHC Key Laboratory of Combined Multi-organ Transplantation; 3 Key Laboratory of the diagnosis and treatment of organ Transplantation, Research Unit of Collaborative Diagnosis and Treatment For Hepatobiliary and Pancreatic Cancer, Chinese Academy of Medical Sciences (2019RU019); 4 Key Laboratory of Organ Transplantation, Zhejiang Province, Hangzhou 310003, China

**Keywords:** the Warburg effect, DNA methylation, aerobic glycolysis, mitochondria, reactive oxygen species

## Abstract

Significant enhancement of the glycolysis pathway is a major feature of tumor cells, even in the presence of abundant oxygen; this enhancement is known as the Warburg effect, and also called aerobic glycolysis. The Warburg effect was discovered nearly a hundred years ago, but its specific mechanism remains difficult to explain. DNA methylation is considered to be a potential trigger for the Warburg effect, as the two processes have many overlapping links during tumorigenesis. Based on a widely recognized potential mechanism of the Warburg effect, we here summarized the relationship between DNA methylation and the Warburg effect with regard to cellular energy metabolism factors, such as glycolysis related enzymes, mitochondrial function, glycolysis bypass pathways, the tumor oxygen sensing pathway and abnormal methylation conditions. We believe that clarifying the relationship between these different mechanisms may further help us understand how DNA methylation works on tumorigenesis and provide new opportunities for cancer therapy.

## Introduction

The Warburg effect, also called aerobic glycolysis, was first discovered by Otto Warburg in the 1920s 1. He observed that tumors required extremely high levels of glucose compared with surrounding normal tissues, and that glycolysis was significantly enhanced tumors, even in the presence of adequate oxygen 2.

It is apparent that the Warburg effect is beneficial to the proliferation and survival of tumor cells, but how it works remains unclear. Based on current opinions, we summarize five major factors that lead to the Warburg effect: (I) The need for rapid ATP synthesis. The energy demands of tumor cells increase rapidly, and the rates of glucose uptake and metabolism through aerobic glycolysis are much higher than those that can be achieved through oxidative phosphorylation (OXPHOS) alone 3. Mitochondrial defects are thought to be another important feature of the Warburg effect, although some researchers disagree. (II) The need for extensive biosynthesis 4-9. Uncontrollably proliferating tumor cells require extensive biosynthesis, and glycolysis and its bypass route - the pentose phosphate pathway (PPP) produce large amounts of raw materials. (III) The need for redox balance in tumor cells. The OXPHOS pathway is one of the main sources of reactive oxygen species (ROS) production, which can cause devastating damage to tumor cells 10. (IV) Stimulation by hypoxia. The accumulation of hypoxia-inducible factor (HIF) leads to deficiency in the aerobic respiratory response and to the activation of glycolysis in tumor cells. (V) The need for an acidic tumor microenvironment 11,12.

DNA methylation is a form of epigenetic modification of gene expression. In mammalian cells, DNA methylation commonly involves the addition of a methyl group contributed by S-adenosy-L-methionine (SAM) to CpG dinucleotides to create 5-methylcytosine (m^5^c), which is catalyzed by DNA methyltransferases (DNMTs)^13^. CpG islands (CGIs), which are characterized by a very high CpG densities and are often found in the promoter regions of genes, are typically hypomethylated. Methylation of CGIs results in transcriptional silencing. In normal cells, methylation ensures the proper gene expression regulation and stable gene silencing, but abnormal DNA methylation is a powerful cause of many tumors. Unlike that of DNA methylation, the mechanism of DNA demethylation has not been well elucidated. Studies have shown that ten-eleven translocation methylcytosine dioxygenases (TETs) can oxidize m^5^c to 5-hydroxymethylcytosine (hm^5^c) 14. hm^5^c and its further oxidized derivatives are subsequently replaced with an unmodified cytosine by base-excision repair to achieve demethylation 15. In myeloid leukemia and glioblastoma cells, inhibition of TETs enzymes decreases the levels of hm^5^c increases DNA methylation 15-18.

**Figure 1:**
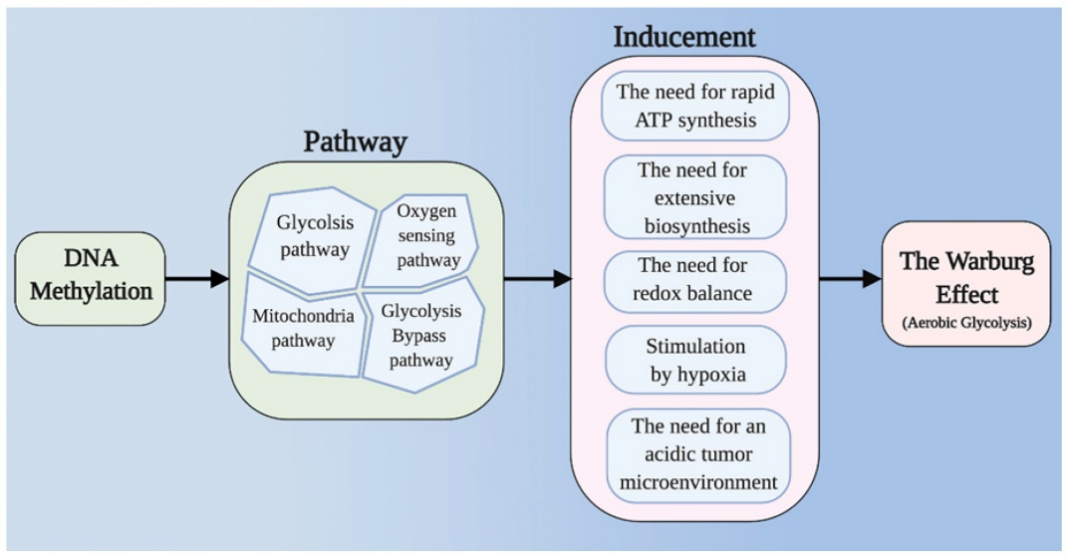
Main framework. The need for rapid ATP synthesis, biosynthesis, maintainance of redox balance and acidification of the tumor microenvironment are all internal causes of the Warburg effect, and HIF accumulation is an important extrinsic cause. Through different pathways (including the glycolysis pathway, the mitochondrial pathway, the glycolysis bypass pathway, and the oxygen sensing pathway), DNA methylation can affect these causes of the Warburg effect.

It is frequently reported that changes in DNA methylation levels regulate the expression of key enzymes in glycolysis. DNA methylation is also reported to cause mitochondrial dysfunction in tumor cells. Tumor redox balance and the accumulation of HIF have also been widely reported to be associated with aerobic glycolysis, and DNA methylation has been found to play a regulatory role in these processes. The PPP and gluconeogenesis, as important bypass pathways of glycolysis, provide abundant raw materials for the rapid proliferation of tumor cells; DNA methylation can participate in the regulation of key enzymes and molecules in these processes, thus playing a potentially important role in aerobic glycolysis in tumor cells. In this review, we discuss how DNA methylation contributes to tumor aerobic glycolysis in different pathways (Figure 1) (Table 1).

## DNA methylation regulates enzymes involved in glycolysis

The main feature of the Warburg effect is that cytoplasmic metabolism becomes the main source of energy instead of mitochondrial aerobic respiration. Although the amount of ATP synthesized by glycolysis per unit of glucose is small 19,20, the ATP synthesis rate is 10-100 times faster than that of complete oxidation in mitochondria; Thus, glycolysis can meet the rapid energy requirements of tumor cells 21. DNA methylation can directly regulate glycolysis by affecting key enzymes in glycolysis.

### Glucose transporter (GLUT)

Due to their rapid proliferation, malignant tumors require enormous amounts of glucose to meet their energy metabolism and anabolism needs. GLUT, a member of the glucose transporter family, is the first key molecule in tumor glucose metabolism. Upregulation of GLUT1 enhances glucose entry into tumor cells, which promotes aerobic glycolysis. Previous studies have shown the relationships between GLUT1 and poor prognosis and aggressiveness in many kinds of cancer, such as breast, kidney and stomach 22-25. Lopez-Serra et al. demonstrated the occurrence of promoter CGI hypermethylation-linked inactivation of Derlin-3 (DERL3), a key gene in the endoplasmic reticulum-associated protein degradation pathway. The downstream targets of DERL3 include GLUT1, which means DERL3 is responsible for the degradation of GLUT1. Increases in the expression of GLUT significantly increase the uptake and transport of glucose and ultimately promote aerobic glycolysis. The final metabolites, lactate and pyruvate, acidify the tumor microenvironment and enhance tumor proliferation and invasion 26,27. In addition to GLUT1, GLUT3 has also been reported to be related to DNA methylation. Sung et al. discovered a structural protein—caveolin-1(CAV-1), whose expression is reduced in the contexts of many human cancers 28. With the promoter CpG site hypomethylation, the expression of CAV-1 is abnormally elevated, which stimulates GLUT3 transcription via the high-mobility group protein A (HMGA) binding site within the GLUT3 promoter, thus, upregulating glucose uptake and ultimately enhancing aerobic glycolysis 29.

### Lactate dehydrogenase (LDH)

LDH, a crucial enzyme for aerobic glycolysis, converts pyruvate into lactate and oxidizes NADH to regenerate NAD+. LDH can guarantee the sustained utilization of NADH-generating glyceraldehyde-3-phsophate dehydrogenase (GAPDH) and maintain orderly aerobic glycolysis. LDH is a tetrameric enzyme consisting of 2 major subunits (A and B), that are encoded by 2 different genes, LDH-A and LDH-B 30. Different numbers of LDH-A and LDH-B subunits can bind in tetramers to form 5 different isoenzymes (LDH-1 to LDH-5) 31. As LDH-5 consists only of LDH-A and preferentially catalyzes the conversion of pyruvate into lactate, whereas LDH-1 consists only of LDH-B and catalyzes the conversion of lactate into pyruvate, the LDH-A/LDH-B ratio in tumor cells modulates lactate production 31. Although HIF-1α- and c-Myc-related pathways can promote LDH-A expression 30, many studies have demonstrated that DNA methylation regulates the LDH-A ratio by promoting LDH-B promoter region hypermethylation in breast and prostate cancer, while the demethylating agent 5-azacytidine can restore the mRNA levels 13. Another study has reported that LDH-A is silenced in isocitrate dehydrogenase (IDH) mutant gliomas because of hypermethylation 32. As the LDH-A/LDH-B ration increases, it greatly promotes the conversion of lactate, which enhances aerobic glycolysis and tumorigenesis 33.

### Pyruvate kinase (PK)

PK, a key rate-limiting enzyme in the glycolysis pathway 34, catalyzes the conversion of phosphoenolpyruvate and ADP to pyruvate and ATP. Because of mutually exclusive alternative splicing 35, PK is divided into two types 36; the alternatively spliced isoform M2 (PKM2) contributes to the Warburg effect by promoting aerobic glycolysis, whereas the PKM1 isoform promotes OXPHOS. Some believe that PKM2 is often upregulated in tumors due to intron hypomethylation, and hence promotes the Warburg effect 13. However, others have suggested that PKM2 stimulates the Warburg effect because of DNA methylation. Singh et al. reported that the intragenic DNA methylation-mediated binding of Brother of Regulator of Imprinted Sites (BORIS) at the alternative exon 10 of PK is associated with cancer-specific splicing that promotes the Warburg effect and breast cancer progression. Once DNA methylation is inhibited or the BORIS binding site is deleted, a splicing switch from the cancer-specific PKM2 to the normal PKM1 isoform occurs. In this case, glycolysis and the Warburg effect are inhibited, limiting rapid proliferation and growth of tumor cells 37. In the same year, Xu et al. proposed another explanation based on mitochondria function related to the effects of coactivator-associated arginine methyltransferase 1 (CARM1), which interacts with and methylates PKM2 at three arginine residues (R445, R447 and R455). Methylated PKM2 localizes to the mitochondria-associated endoplasmic reticulum membrane, through interaction with inositol 1,4,5-trisphosphate receptors (IP3Rs), decreasing mitochondrial membrane potential (ΔΨm) and Ca2+ uptake, which is essential for activating pyruvate dehydrogenase (PDH) to support OXPHOS and thus ultimately promoting aerobic glycolysis^38^. Hence, PKM2 methylation is an important regulator of the switch between OXPHOS and aerobic glycolysis in cancer cells. PKM2 can also further promote the aerobic glycolysis process in tumor cells by affecting the PPP, which will be described later. Therefore, the specific mechanism and status of PKM2 methylation in tumor cell metabolism are still worth studying.

### Hexokinases (HKs)

HKs catalyze the first step in glycolysis, the ATP dependent phosphorylation of glucose to yield glucose-6-phosphate (G6P). Four major hexokinase isoforms, encoded by separate genes, are expressed in mammalian tissues: HK1, HK2, HK3, and HK4. HK2 is often expressed at high levels in tumor cells 39,40. Hypomethylation of the promoter is responsible for the upregulation of HK2 in liver cancer and glioblastoma 41; this upregulation then enhances aerobic glycolysis and tumor proliferation 42. HK2 has also been found to be mediated by microRNAs in ovarian cancer, such as miR603 and miR145 43,44, whose precursor genes are modulated by DNMT3a. Given these findings, microRNAs can be considered inhibitors of the Warburg effect. And the inhibitory effects of miR603 and miR145 can be enhanced by the use of 20(S)-Rg3, an inhibitor of DNMT3a.

### GAPDH

GAPDH converts glyceraldehyde-3-phosphate (G3P) and NAD+ into 1,3-bisphosphoglycerate (1,3-BPG) and NADH. It also plays an important role in aerobic glycolysis and has been found to be upregulated in the contexts of several kinds of cancer, but the exact cause of the upregulation remains unclear. Recently, Lei et al. proposed a new explanation for how GAPDH functions in the Warburg effect 45. They focused on CARM1, which methylates GAPDH at R234 and inhibits its catalytic function in hepatocellular carcinoma (HCC). Therefore, aerobic glycolysis was repressed and the proliferation and growth of HCC cells are repressed.

## Mitochondrial dysfunction caused by DNA methylation is a potential trigger for aerobic glycolysis

**Figure 2:**
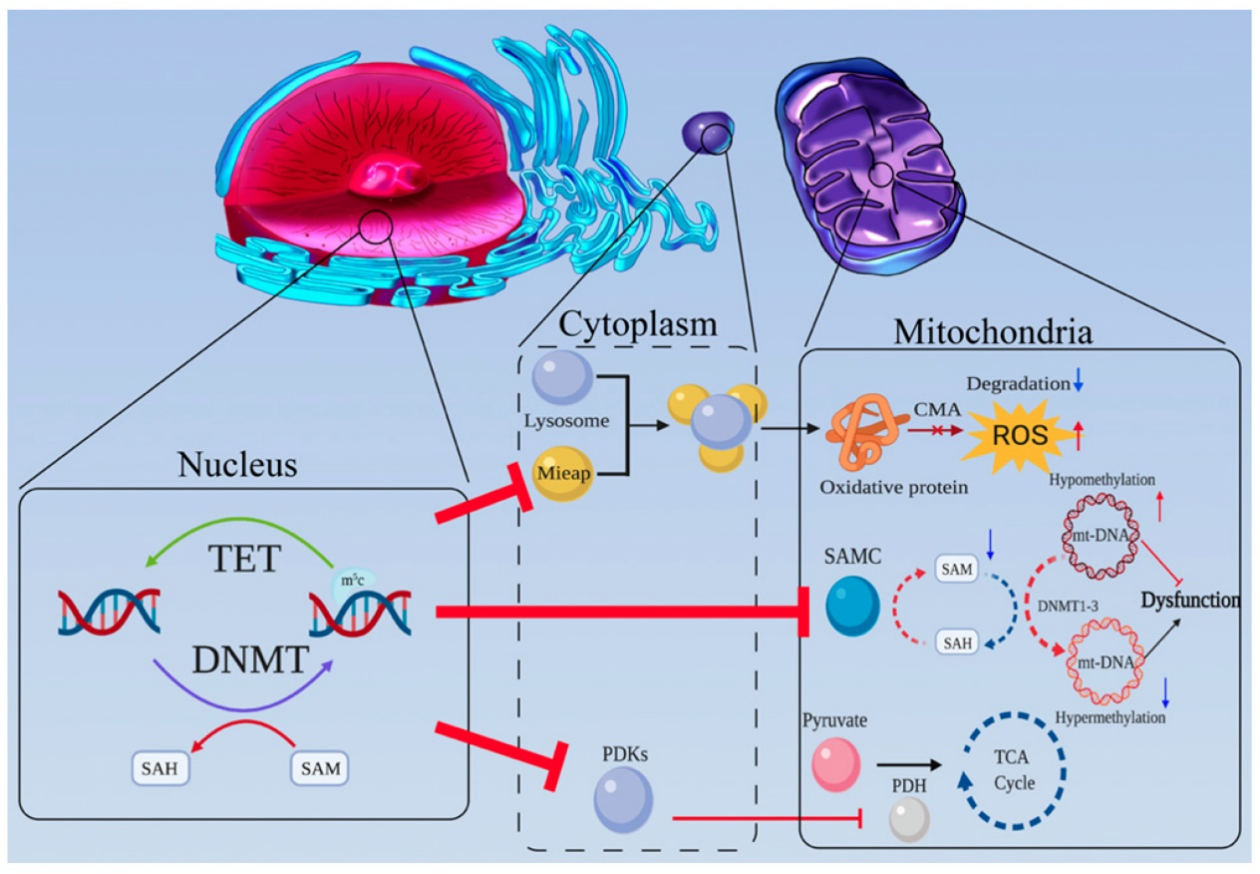
Mitochondrial dysfunction caused by DNA methylation is a potential trigger for aerobic glycolysis. **(a)** MtDNA is crucial for mitochondrial function. Once mtDNA is methylated by mt-DNMTs, OXPHOS is inhibited. Due to promoter hypermethylation, SAMC is downregulated, and mitochondrial SAM levels decrease. Hence, the methylation status of mtDNA may be downregulated. **(b)** Mieap-mediated, lysosomal involved oxidative protein clearance is an important pathway for the maintenance of normal mitochondrial function. Hypermethylation of Mieap causes mitochondria to become severely dysfunctional. **(c)** The activity of PDH can be inhibited by PDKs, which are modulated by DNA methylation.

Mitochondria are the most important organelles for cell energy metabolism. As the end product of glycolysis, pyruvate enters the mitochondria for further reactions to produce ATP and metabolites through the tricarboxylic acid (TCA) cycle and OXPHOS. Abnormalities in the structure or function of mitochondria are very common in tumors, such as mitochondrial DNA (mtDNA) damage in tumor cells, which affects mitochondrial respiration and energy synthesis 46,47. There is abundant evidence suggesting mitochondrial dysfunction is a potential trigger for aerobic glycolysis 48-50 (Figure 2).

### Abnormalities in mtDNA

MtDNA accounts for only a very small proportion of the total cellular DNA 51-53, but its gene expression products play crucial roles in mitochondrial and cellular functions. MtDNA encodes several important proteins related to the mitochondrial respiratory chain 54,55. Therefore, changes in mtDNA status will directly affect mitochondrial function and ultimately glycolysis status. Whether methylation of mtDNA exists has long been debated in the growing field of tumor metabolism research 56-60. Shock et al. first reported the presence of m5c and hm5c in mitochondria which might be derived from mtDNA cytosine methylation, and the existence of mitochondrial DNA methyltransferase 1 (mt-DNMT1) which bound to mtDNA and modifies the mitochondrial genome and function 61. Sunil et al. further identified the DNMT1 in mitochondria as isoform 3 62. Marianne et al. first directly proved the potential functionality of this molecule in tumor cells 63 and some other studies have also suggested a direct relationship between mtDNA methylation and tumors 64,65. Notably methylation of mtDNA generally leads to OXPHOS dysfunction 66. The methyl donor SAM is also important to mtDNA methylation 67. The SLC25A26 gene encodes the SAM mitochondrial carrier (SAMC) which catalyzes the import of SAM into mitochondria 68. In cancer cells, upexpression of SAMC increases mitochondrial SAM levels, promotes mtDNA methylation, leads to decreased expression of key respiratory subunits, and decreases the release of cytochrome B, thus impairing mitochondrial OXPHOS and ROS balance 69. The glycolysis function then is further strengthened for compensation in tumor cells, even in the presence of oxygen.

### Abnormalities in mitochondrial health

Another cause of mitochondrial dysfunction is the accumulation of unhealthy mitochondria. The mitochondrial quality control protein—Mieap can induce intramitochondrial lysosome-like organella that plays a critical role in eliminating oxidized mitochondrial proteins in mitochondria 70,71. It dramatically induces the accumulation of lysosomal proteins in mitochondria without destroying the mitochondrial structure, leading to increased ATP synthesis and decreased ROS generation. 72. An existing model for the mechanism by which Mieap induces lysosome-like organelles enter into mitochondria is chaperon-mediated autophagy (CMA) way, one of the three main pathways of autophagy. In this mechanism, lysosomes can specifically absorb oxidized proteins containing a common motif through the interaction of lysosome-associated membrane protein 2A (LAMP2A) and heat shock protein 70 (HSP70) 73. If entire structure of lysosomes or lysosomal like organelles can enter and reside in the mitochondria, these organelles may act though a CAM-like mechanism in the mitochondria to eliminate and degrade oxidative proteins. The exact mechanism by which Mieap helps lysosomes enter the mitochondria without damaging normal mitochondrial structures remains unclear. However, in many kinds of tumor cell lines, Mieap expression is often inhibited by promoter methylation, leading to ROS accumulation, protein oxidation and subsequent mitochondrial destruction 74.

### Abnormalities in key TCA cycle enzymes

The TCA cycle is the first reaction cycle undergone by glycolysis products in the mitochondria. Abnormalities in TCA enzymes lead to mitochondrial dysfunction and are potential triggers of aerobic glycolysis. PDH mainly mediates the entry of pyruvate into the TCA cycle, while the family of PDH kinases (PDKs) inhibits PDH activity to promote aerobic glycolysis and tumor progression 75,76. Leclerc et al. observed that PDH kinase 4 (PDK4) is distinctly upregulated by hypomethylation of its promoter 77. In addition, 2-hydroxyglutarate (2-HG) produced by IDH1/2 mutation 32, as well as the accumulation of succinate and fumarate caused by mutations in fumarate hydratase (FH) 78 and succinate dehydrogenase (SDH), can not only promote the Warburg effect by inhibiting the degradation of HIF 79, but also enhance the methylation level of the whole genome by inhibiting TET enzymes 80.

## DNA methylation participates in the glycolysis bypass pathways

Tumor cells are rapidly and malignantly proliferating cells, that require many raw materials to support their continuous proliferation. Hence, Heiden et al. proposed another explanation for the Warburg effect: proliferating cells have important metabolic requirements that extend beyond ATP 81. The PPP, a glycolysis bypass route, plays an important role in the production of raw material production, such as nucleic acid and amino acid sugar phosphate precursors. Moreover, the Warburg effect is also affected by changes in the expression levels of PPP enzymes in the nonoxidative pathway.

Transketolase (TKT) like-1(TKTL1) is an essential gene that encodes an enzyme responsible for the TKT reaction in nonoxidized portion of PPP. There is considerable evidence that tumor cells can upregulate the activity of TKT 82 and induce the expression of TKTL1, whose expression has been shown to be increased due to promoter hypomethylation^83^. Wenyue et al. showed that high expression of TKTL1 in tumor cells significantly increases the production of lactate and pyruvate, which are hallmarks of the Warburg effect 84. In addition, they demonstrated that TKTL1 promotes the stability and accumulation of HIF1-α, which is a key molecule for DNA methylation and aerobic glycolysis, that will be discuss later.

Nuclear factor erythroid 2 (NF-E2)-related factor 2 (Nrf2) is a master transcriptional activator of cyto-protective genes 85,86. Its main function is protecting cells from the effects of allogeneic organisms and oxidative stress. However, high Nrf2 expression in tumor cells is often associated with poor prognosis 87,88. Mitsuishi et al. analyzed Nrf2 from the perspective of its effects on tumor metabolism 89. Nrf2 directly activates glucose 6-phosphate dehydrogenase (G6PDH), PGD, TKT, transaldolase and IDH, which are key enzymes of PPP, through well-conserved antioxidant response elements (AREs). Thus, Nrf2 has been shown to promote NADPH and nucleotide production in tumor cells 90. Under normal conditions, Nrf2 is continuously degraded in a keap1-dependent manner through the ubiquitin-proteasome pathway 91,92. Upon the increased methylation in the promoter region, the expression level of keap-1 is significantly decreased, which activates Nrf2 and PPP enzymes, as has been confirmed in lung cancer cells and glioma cells 93,94.

Most studies tend to the catabolism of glucose, ignoring the role of anabolism in aerobic glycolysis. Gluconeogenesis, a process that converts a variety of non-sugar substances into glucose or glycogen, is less investigated than glycolysis but may play an equally important role in switching the metabolism of tumor cells to aerobic glycolysis. Because glycolysis involves a three-step irreversible reaction, gluconeogenesis is not a simple reversal of glycolysis. Notably, the gluconeogenesis pathway can be modulated by DNA methylation. Cai et al. reported that betaine, a methyl donor, can significantly change the methylation statuses of CpGs in the promoters of gluconeogenesis-associated genes 95.

Fructose-1,6-bisphosphatase (FBP) plays a crucial role in the process of gluconeogenesis which catalyzes the hydrolysis of fructose-1,6-bisphosphate into fructose-6-phosphate and inorganic phosphate. There are two isoforms of FBP in humans: FBP1 and FBP2. FBP1 is widely reported to be downregulated, due to abnormal methylation of its promoter sequence in non-small cell lung cancer (NSCLC) 96, HCC 97, basal-like breast tumor 98, gastric cancer 99, small intestinal neuroendocrine tumor 100 and colon cancer 101. Although studies on FBP2 have rarely been reported, hypermethylation in the FBPs promoter region has been shown to occur in gastric cancer cells 102.

In addition, FBP1 has been reported to be a tumor suppressor that regulates tumor glucose metabolism and inhibits aerobic glycolysis, such as by increasing glucose uptake and macromolecules biosynthesis 98,103. FBP1 has also been demonstrated to be involved in posttranslational modification of PKM2 which is a crucial glycolytic enzyme 104-106. Koeck et al. found that upregulation of FBP1 significantly increases mitochondrial complex I activity. In contrast, loss of FBP1 inhibits oxygen consumption and ROS production 107. Li et al. reported that ectopic FBP1 expression in renal cell carcinoma reduces lactate secretion, NADPH level, PPP flux and glycolysis derived TCA cycle intermediates levels 108. These data provide mechanistic insights that loss of FBP caused by DNA methylation may result in glycolytic flux, glucose uptake, ATP production maintenance 98,109, and OXPHOS functional inhibition 98, all of which are features of the Warburg effect.

FBP, a key enzyme of gluconeogenesis pathway, mainly converts lactate and pyruvate into glucose for reuse, which is beneficial to cells. However, in tumor cells, FBP acts as a tumor suppressor and inhibits aerobic glycolysis. We hypothesize that FBP can promote glycolytic flux into the gluconeogenesis pathway and destroy the acidic environment. Moreover, gluconeogenesis, the reverse pathway of glycolysis, requires much more energy than glycolysis since it must overcome the three-step irreversible glycolysis reaction 110,111, which is not efficient for smart tumor cells.

## Oxygen-sensing pathway connects DNA methylation and aerobic glycolysis

**Figure 3:**
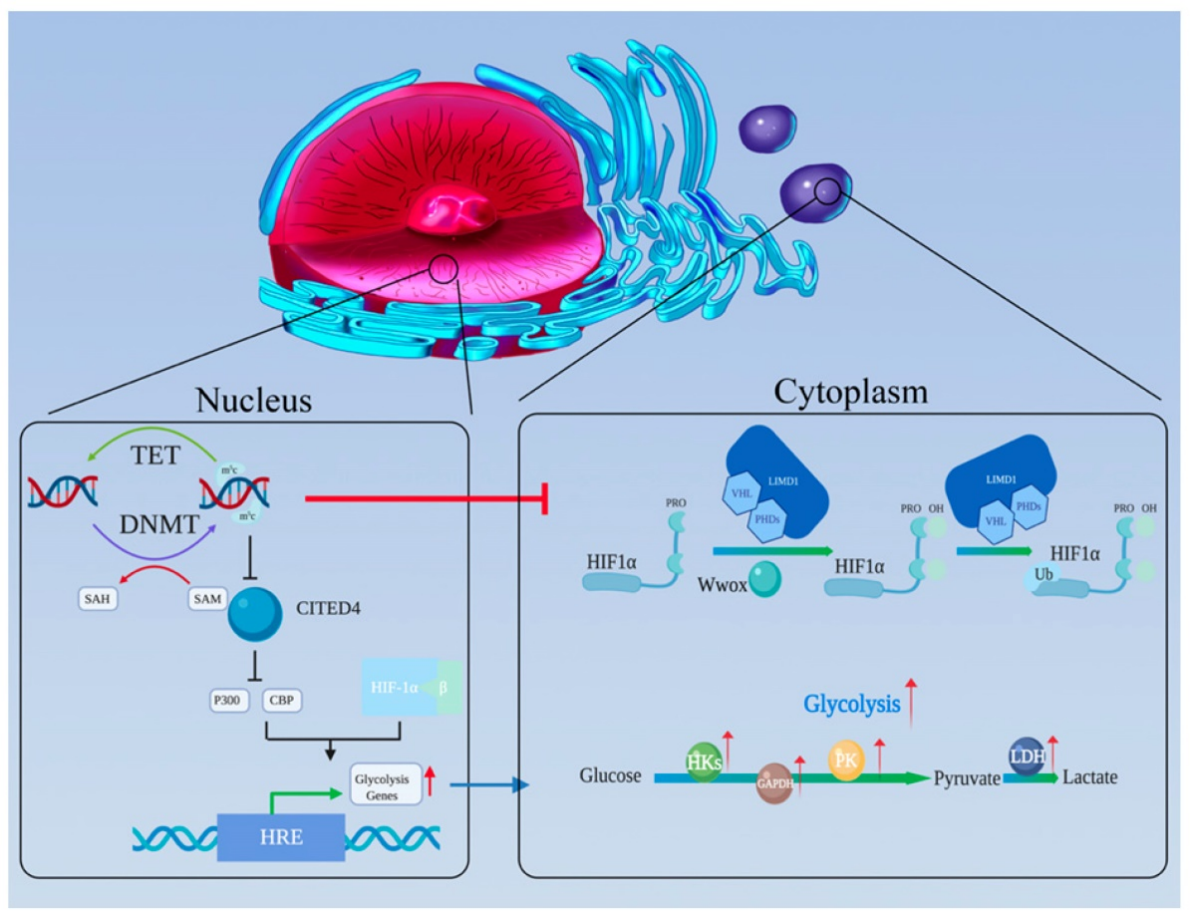
The oxygen-sensing pathway connects DNA methylation and aerobic glycolysis. **(a)** The HIF complex can recruit P300 and CBP and then stimulate the HREs in the promoters of its target genes, promoting glycolysis-related gene expression. **(b)** CITED4 can competitively bind P300 and CBP, thereby inhibiting HIF's function. However, CITED4 is regulated by DNA methylation. **(c)** Wwox, which is regulated by DNA methylation, could disrupt HIF1-α's stability by affecting the PHD pathway. **(d)** LIMD1 acts as a scaffold to bind PHDs and VHL, which are key molecules for the degradation of HIF1-α. LIMD1 and VHL can be inhibited by DNA methylation.

As the central protein of the hypoxia regulatory pathway, HIF has been demonstrated to be associated with the Warburg effect. HIF consists of oxygen-related α-subunits (HIF1-α and HIF2-α) and a constitutively expressed β-subunit (HIF1-β) 112. Among the subunits, HIF1-α is believed to be the one most related to tumor glycolysis 113. Under conditions of normal oxygen, a-ketoglutarate-dependent prolyl-hydroxylases (PHDs) promotes the hydroxylation of HIF-α proline residues 114-116, which becomes the optimal recognition sites for the von Hipppel-Lindau (VHL) tumor suppressor protein. VHL binding activates ubiquitination pathways to degrade HIF 117-119. Hydroxylation requires oxygen and α-ketoglutarate, and produces carbon dioxide and succinate. In the absence of oxygen, PHD activity is inhibited, and the HIF-α subunit is no longer degraded; rather it binds to the constitutively expressed HIF-β subunit to form a HIF dimer. HIF dimer then enters the nucleus and binds to hypoxia response elements (HREs) (Figure 3) 120-122.

The presence of hypoxic regions is characteristic of the microenvironment of solid tumors, such as liver cancers 123. HIF regulates many genes in multiple cell types, including those related to glycolysis 124. This regulation allows tumor cells to transfer the center of energy metabolism from OXPHOS to glycolysis under hypoxic condition 125. Many studies have demonstrated that the expression levels of glycolysis related genes such as 6-phosphofructto-1-kinase (PFK-1), PKM2 and 6-phosphofructo-2-kinase/fructose 2,6-bisphosphatase genes (PFKFB1-4), are increased in tumor cells under hypoxia condition^126^,^127^. Keith B et al. reported that elevated HIF activity stimulates the expression of glycolysis-related genes, such as LDHA, phosphoglycerate kinase 1 (PGK1), and activated PDH kinase 1(PDK1) to inhibit glycolytic flux into the TCA cycle in clear cell renal cell carcinoma (ccRCC) 128. Bo Li et al. found that FBP1 suppresses HIF activity and eventually reduces the expression of HIF target genes (PDK1, LDHA and GLUT1) 103.

Hu et al. first demonstrated that HIF1-α and HIF2-α have unique targets, and that HIF1-α (not HIF2-α) stimulates glycolysis-related gene expression. Swati Dabral et al. found that Ras association domain family 1A (RASSF1A) can bind to HIF1-α, block its degradation in the PHD-VHL-lysosome pathway, and thus enhance the activation of the glycolytic switch in lung cancer cells 129. HIF-1α can also inhibit mitochondrial function and thus promote aerobic glycolysis. After entering the mitochondria, pyruvate dehydrogenase (PDH) catalyzes the conversion of pyruvate into acetyl coenzyme A and enters the TCA cycle. HIF-1α can activate PDK1 (phosphatidylinositol-dependent protein kinase 1), which can then phosphorylate and inhibit pyruvate dehydrogenase (PDH) E1α^130^, thus inhibiting acetyl coenzyme A synthesis, and blocking the TCA cycle and thereby freeing pyruvate from mitochondrial OXPHOS. Therefore, we can conclude that HIF is closely related to glycolysis.

The finding described above reveal that HIF plays an important role in aerobic glycolysis. In addition, there is considerable evidence that HIF can be regulated by DNA methylation (Figure 3). M Koslowski et al. first revealed the relationship between tumor-associated CpG demethylation and HIF-1α. In colon cancer cell lines, treatment with the DNA-demethylating agent 5-azacitidine significantly enhances the expression of HIF-1α its target genes. The potential mechanism involves positive autoregulation of HIF-1α is governed by a methylation-sensitive HRE in its promoter 131.

Furthermore, DNA methylation is involved in regulating the functional pathways of HIF. After HIF-1α and HIF-β combine to form a stable dimer and translocate to the nucleus, the HIF dimer needs to recruit the transcriptional adapter/histone acetyltransferase protein, P300 and CREB-binding protein (CBP), to the promoter of its target genes for transcription stimulating 132,133. Carboxy-terminal domain 4 (CITED4) could competitive bind P300/CBP and inhibit the HIF complex. Due to hypermethylation of its promoter, the expression of CITED4 is inhibited in breast cancer, while that of HIF and its target genes is significantly increased 134.

In addition, DNA methylation regulates HIF degradation pathways. As a tumor suppressor, WW-domain containing oxidoreductase (Wwox) has been reported to modulate glucose metabolism 135. Abu-Remaileh M et al. found under aerobic conditions, Wwox loss activates glycolysis-related gene expression and inhibits pyruvate entry into the TCA cycle which are features of the Warburg effect 136. The authors explained that these effects happen because Wwox disrupts HIF1-α's stability via affecting the PHD pathway and inhibiting its transcriptional activity 137. Ekizoglu S.et al. found that the expression level of Wwox and the methylation of its promoter are inversely correlated. In other words, Wwox expression is regulated by DNA methylation 138. The tumor suppressor protein LIM domain containing protein (LIMD1) has been demonstrated to act as a scaffold to bind PHDs and VHL, which are responsible for HIF degradation 139. And Panda CK et al. showed that upregulation of HIF1-α and its target genes was due to high methylation status of LIMD1 and VHL in cervical carcinoma 140.

Although HIF1-α is often thought to be associated with glycolysis, HIF-2α has also been showed to regulate GLUT-1, which could enhance aerobic glycolysis and glucose transport into renal carcinoma cells 141. Some research has shown that a target gene of HIF2-α, endothelial PAS domain-containing protein 1 (EPAS1), is modulated by DNA methylation. Blockade of the transcription of EPAS1 by DNMT3a can inhibit tumor proliferation in renal cell carcinoma and glioblastoma cell lines 142,143.

## Abnormal conditions related to DNA methylation

The methyl donor SAM is a key factor for DNA methylation. The main principle of DNA methylation is the addition of a methyl group contributed by SAM to CpG dinucleotides to create m5c. Folate is an important source for SAM synthesis. After absorption in the small intestine, folate is converted into its active form, tetrahydrofolate (THF), which then combines with one carbon unit to form methylene THF. It is a raw material for DNA and RNA synthesis. Reductase converts methylene THF into 5-methyl-THF, which is combined with homocysteine (Hcy) to form methionine; methionine then is converted to SAM 144.

Low-folate nutritional status (LF) has been reported to play a roles in lung cancer 145 and colon cancer 146. LF leads to low SAM expression, which reduces the methylation levels in tumor cells, thereby changing the functions of tumor cells. In addition, Jin Fan et al. first demonstrated that folate metabolism is a crucial method for NADPH production; these authors knocked down the folate-dependent enzymes, methylenetetrahydrofolate dehydrogenase (MTHFD), which significantly reduced NADPH/NADP+ ratios 147. In addition, MTHFD expression has been found to be related to the progression of cancers 148. It has even been shown that the folate pathway produces more NADPH than the PPP. Roland Nilson et al. reported that MTHFD2 is overexpressed in 12 tumor types 149. Under LF, NSCLCs have been reported to undergo metabolic reprogramming, which includes elevation in lactate release, acidification of the microenvironment, the change in the NADH/NAD+ redox status and NADPH/NADP+ ratios. Changes in these characteristics will inhibit aerobic glycolysis. And The LF-induced aerobic glycolysis phenotype of NSCLC can be reversed by the DNA methylation inhibitor 5-azacitidine. This result suggests that the LF status promotes the Warburg effect by reducing methylation levels in tumor cells 150. The methionine salvage pathway is another crucial pathway for SAM production 151,152. Serine metabolism was thought to promote the methionine salvage pathway, a bypass pathway for glycolysis, through a range of anabolic processes, including NADPH, methylene THF and nucleotides 153. These metabolites are all important intermediates for the methionine salvage pathway to promote SAM and DNA methylation.

## Future perspectives

The synergistic effect of The Warburg effect and DNA methylation is worthy of further study, which is conducive to further revealing the mechanism of tumor development and tumor therapy.

### The synergistic effects for tumor immunity

Aerobic glycolysis and DNA methylation not only work together to regulate tumor cells directly, but also may influence the development of tumors by regulating the function of the immune system. Aerobic glycolysis is considered to be a metabolic hallmark of activated T cells. Pearce EL et al. suggested that aerobic glycolysis augments effector T cell responses, including expression of the proinflammatory cytokine interferon (IFN)-γ via 3′ untranslated region (3′UTR)-mediated mechanisms 154. Li et al. found that aerobic glycolysis can promote effector T cell differentiation 155. While GA et al. reported that DNMT3a can also regulate T cell development and suppress T-cell acute lymphoblastic leukemia transformation 156. Hence, further studying DNA methylation and aerobic glycolysis in immune cells could be of great significance for enhancing understanding of tumor immune tolerance.

### The synergistic effects for tumor therapy

Both DNA methylation and the Warburg effect are important mechanisms of tumor development and provide us with new strategies for tumor treatment. The Warburg effect provides advantages for the growth of tumor cells; therefore, some drugs can alleviate mitochondrial OXPHOS defects and inhibit glycolysis by regulating the energy acquisition pathways of cells. For example, 2-deoxy-d-glucose (2-DG), a glucose analog, affects glucose metabolism, depleting cancer cells of energy and eliciting antitumor effects 157. Dichloroacetic acid (DCA) can reverse the Warburg effect by inhibiting PDK1 to switch cytoplasmic glucose metabolism to OXPHOS, providing a new approach to antitumor therapy 158. 3-Bromopyruvate (3-BP) is also a widely recognized inhibitor of glycolysis 159. As a trigger of the Warburg effect, ROS are produced mainly through mitochondria. Strategies for mitochondrial metabolism have been reported in many clinical studies 160. In addition to limitation of aerobic glycolysis, inhibition of DNA methylation is also considered an important approach for cancer therapy. The DNA methylation inhibitors decitabine (5-aza-2'-deoxycytidine) and 5-azacitidine have been widely used in the treatment of leukemia 161.

Many studies have shown that the regulation of ROS and DNA methylation play a synergistic role in the treatment of tumors. Poly (adp-ribose) polymerase (PARP) inhibitors (PARPi) are an effective anti-tumor drug for breast and ovarian cancer. And DNA methylation inhibitor decitabine can mediate the activation of PARP by increasing the accumulation of ROS, and promote the sensitivity of PARPi to tumors through the cAMP/PKA pathway, so as to play a more effective anti-tumor effect in collaboration with PARPi^162^. Zhou et.al proposed that Live-attenuated measles virus vaccine as a potential oncotherapeutic agent, confers cell contact loss and apoptosis of ovarian cancer cells via ROS-induced silencing of E-cadherin by DNA methylation 163. Deepika et.al reported a special hypoxia-selective epigenetic agent RRx-001, which induces reactive oxygen species and nitrogen (RON), and in turn induces oxidative and nitrogen narrative stress, leading to cell death in myeloma. RRx-001 also inhibits DNA methylation by down-regulating DNA methyltransferase (DNMTs) and induces tumor cell apoptosis 164. These suggest that combining multiple mechanisms to treat tumors may yield better results.

Elucidating the relationship between various important tumorigenesis mechanisms, finding out the root cause of dysfunction, and developing new combination treatment will be the development direction of tumor therapy.

## Conclusion

DNA methylation, a type of epigenetic modification, plays an important role in both normal and tumor cells. The Warburg effect, a characteristic marker of abnormal metabolism in tumor cells, warrants further study. In this review, we have summarized the correlations between DNA methylation and the Warburg effect, and have discussed the mechanism by which DNA methylation may contributed to the Warburg effect. DNA methylation can regulate glycolysis related enzymes, inhibit mitochondrial functions and glyconeogenesis-related enzymes, promote aerobic glycolysis, enable the rapid energy needs of tumor cells to be met and reduce ROS damage. The PPP, which produces NADPH for redox equilibrium and raw material production, can also be modulated by DNA methylation. HIF has been widely reported to promote aerobic glycolysis and is closely associated with DNA methylation. We have also discussed how abnormal DNA methylation conditions can affect aerobic glycolysis, further supporting the important role of DNA methylation in the occurrence and development of Warburg effect. However, as Otis W. Brawley once said, “One cancer cell is smarter than 100 brilliant cancer scientists”. Thus, there is still much to be learned about the association between DNA methylation and the Warburg effect.

## Figures and Tables

**Table 1:** Targets of DNA methylation function in aerobic glycolysis.

Target	Pathway	Inducement	Cancer/cell type	Reference
**GLUT-1**	Glycolysis	Rapid ATP synthesis /Acidic microenvironment	Colorectal cancer	26
**LDH**	Glycolysis	Rapid ATP synthesis / Redox balance/Acidic microenvironment	Breast cancer	13
**PKM2**	Glycolysis / PPP	Rapid ATP synthesis/ Redox balance/Acidic microenvironment	Breast cancer / Pancreatic cancer	37 38
**HK**	Glycolysis	Rapid ATP synthesis /Acidic microenvironment	HCC / Glioblastoma / Ovarian cancer	39 41 42
**GAPDH**	Glycolysis	Rapid ATP synthesis /Acidic microenvironment	HCC	45
**mt-DNA**	Mitochondria	Rapid ATP synthesis / Redox balance	Colorectal cancer	63 64 65
**Mieap**	Mitochondria	Rapid ATP synthesis / Redox balance	Colorectal cancer / Hepatoblastoma	74
**TKTL1**	Bypass pathway (PPP)	Biosynthesis Requirement / HIF accumulation	Head and neck squamous cell carcinoma	82 83
**NRF2**	Bypass pathway (PPP)	Redox balance / Biosynthesis Requirement	Lung cancer cells / Glioma cells	87 88
**FBP1**	Bypass pathway (Gluconeogenesis)	Glycolysis / Redox balance / Acidic microenvironment	NSCLC / HCC/Breast cancer	96 97,98
**Wwox**	Oxygen sensing pathway	HIF accumulation	Head and neck squamous cell carcinoma	138
**PDK**	Mitochondria	Rapid ATP synthesis / REDOX balance	Colorectal cancer	77
**CITED4**	Oxygen sensing pathway	HIF function	Breast cancer	134
**LIMD1**	Oxygen sensing pathway	HIF accumulation	Cervical carcinoma	140

**Abbreviation:** GLUT: glucose transport; LDH: lactate dehydrogenase; PK: pyruvate kinase; PK: pyruvate kinase; GAPDH:glyceraldehyde-3-phosphate dehydrogenase; Mieap: mitochondrial quality control protein; TKTL1: transketolase like-1; Nrf2: NF-E2-related factor 2; Nrf2: NF-E2-related factor 2; FBP: fructose-1,6-bisphosphatase; FBP: fructose-1,6-bisphosphatase; PDK: pyruvate dehydrogenase (PDH) kinase family; CITED4: Carboxy-terminal domain4; HNSCC: Head and neck squamous cell carcinoma; HCC: hepatocellular carcinoma; NSCLC: non-small-cell lung cancer.

## Abbreviations

BORIS: brother of regulator of imprinted sites
CITED4: carboxy-terminal domain 4
CGIs: CpG islands
CAV-1: caveolin-1
CARM1: coactivator-associated arginine methyltransferase 1
CMA: chaperon-mediated autophagy
DNMTs: DNA methyltransferases
DERL3: derlin-3
EPAS1: endothelial PAS domain-containing protein 1
FBP: fructose-1,6-bisphosphatase
GLUT: glucose transporter
G6P: glucose-6-phosphate
GAPDH: glyceraldehyde-3-phosphate dehydrogenase
G3P: glyceraldehyde-3-phosphate
HIF: hypoxia-inducible factor
hm^5^c: 5-hydroxymethylcytosine
HMGA: high mobility group protein A
HREs: hypoxia response elements
HKs: hexokinases
IDH: isocitrate dehydrogenase
LDH: lactic dehydrogenase
LF: low-folate nutritional status
LIMD1: LIM domain containing protein
MTHFD: methylenetetrahydrofolate dehydrogenase
m^5^c: 5-methylcytosine
Mieap: mitochondrial quality control protein
NF-E2: nuclear factor erythroid 2
Nrf2: NF-E2-related factor 2
NADPH: reduced nicotinamide adenine dinucleotide phosphate
PPP: pentose phosphate pathway
PDK1: phosphatidylinositol-dependent protein kinase 1
PGK1: phosphoglycerate kinase 1
PFKFB: 6-phosphofructo-2-kinase /fructose 2,6-bisphosphatase
PFK-1: 6-phosphofructto-1-kinase
PHD: prolyl-hydroxylases
PDH: pyruvate dehydrogenase
PK: pyruvate kinase
ROS: reactive oxygen species
SAM: S-adenosylmethionine
TCA: tricarboxylic acid cycle
TET: ten-eleven translocation methylcytosine dioxygenases
TKTL1: transketolase like-1
THF: tetrahydrofolate
OXPHOS: oxidative phosphorylation
VHL: von Hippperl-Lindau
Wwox: WW-domain containing oxidoreductase
1,3-BPG: 1,3-bisphosphoglycerate

## References

[1] Warburg O, Wind F, Negelein E (1927). THE METABOLISM OF TUMORS IN THE BODY. J Gen Physiol.

[2] WARBURG O (1956). On the origin of cancer cells. Science.

[3] Epstein T, Xu L, Gillies RJ, Gatenby RA (2014). Separation of metabolic supply and demand: aerobic glycolysis as a normal physiological response to fluctuating energetic demands in the membrane. Cancer Metab.

[4] Levine AJ, Puzio-Kuter AM (2010). The control of the metabolic switch in cancers by oncogenes and tumor suppressor genes. Science.

[5] DeBerardinis RJ, Lum JJ, Hatzivassiliou G, Thompson CB (2008). The biology of cancer: metabolic reprogramming fuels cell growth and proliferation. Cell Metab.

[6] Dang CV (2012). Links between metabolism and cancer. Genes Dev.

[7] Koppenol WH, Bounds PL, Dang CV (2011). Otto Warburg's contributions to current concepts of cancer metabolism. Nat Rev Cancer.

[8] Patra KC, Wang Q, Bhaskar PT, Miller L, Wang Z, Wheaton W (2013). Hexokinase 2 is required for tumor initiation and maintenance and its systemic deletion is therapeutic in mouse models of cancer. Cancer Cell.

[9] Boroughs LK, DeBerardinis RJ (2015). Metabolic pathways promoting cancer cell survival and growth. Nat Cell Biol.

[10] Ho PC, Bihuniak JD, Macintyre AN, Staron M, Liu X, Amezquita R (2015). Phosphoenolpyruvate Is a Metabolic Checkpoint of Anti-tumor T Cell Responses. Cell.

[11] Estrella V, Chen T, Lloyd M, Wojtkowiak J, Cornnell HH, Ibrahim-Hashim A (2013). Acidity generated by the tumor microenvironment drives local invasion. Cancer Res.

[12] Gatenby RA, Gawlinski ET (1996). A reaction-diffusion model of cancer invasion. Cancer Res.

[13] Yen CY, Huang HW, Shu CW, Hou MF, Yuan SS, Wang HR (2016). DNA methylation, histone acetylation and methylation of epigenetic modifications as a therapeutic approach for cancers. Cancer Lett.

[14] Tahiliani M, Koh KP, Shen Y, Pastor WA, Bandukwala H, Brudno Y (2009). Conversion of 5-methylcytosine to 5-hydroxymethylcytosine in mammalian DNA by MLL partner TET1. Science.

[15] Shen L, Wu H, Diep D, Yamaguchi S, D'Alessio AC, Fung HL (2013). Genome-wide analysis reveals TET- and TDG-dependent 5-methylcytosine oxidation dynamics. Cell.

[16] Xiao M, Yang H, Xu W, Ma S, Lin H, Zhu H (2012). Inhibition of alpha-KG-dependent histone and DNA demethylases by fumarate and succinate that are accumulated in mutations of FH and SDH tumor suppressors. Genes Dev.

[17] Figueroa ME, Abdel-Wahab O, Lu C, Ward PS, Patel J, Shih A (2010). Leukemic IDH1 and IDH2 mutations result in a hypermethylation phenotype, disrupt TET2 function, and impair hematopoietic differentiation. Cancer Cell.

[18] Xu W, Yang H, Liu Y, Yang Y, Wang P, Kim SH (2011). Oncometabolite 2-hydroxyglutarate is a competitive inhibitor of alpha-ketoglutarate-dependent dioxygenases. Cancer Cell.

[19] Vander HM, Cantley LC, Thompson CB (2009). Understanding the Warburg effect: the metabolic requirements of cell proliferation. Science.

[20] Locasale JW, Cantley LC (2011). Metabolic flux and the regulation of mammalian cell growth. Cell Metab.

[21] Liberti MV, Locasale JW (2016). The Warburg Effect: How Does it Benefit Cancer Cells?. Trends Biochem Sci.

[22] Zhang TB, Zhao Y, Tong ZX, Guan YF (2015). Inhibition of glucose-transporter 1 (GLUT-1) expression reversed Warburg effect in gastric cancer cell MKN45. Int J Clin Exp Med.

[23] Younes M, Brown RW, Stephenson M, Gondo M, Cagle PT (1997). Overexpression of Glut1 and Glut3 in stage I nonsmall cell lung carcinoma is associated with poor survival. Cancer-Am Cancer Soc.

[24] Kawamura T, Kusakabe T, Sugino T, Watanabe K, Fukuda T, Nashimoto A (2001). Expression of glucose transporter-1 in human gastric carcinoma: association with tumor aggressiveness, metastasis, and patient survival. Cancer-Am Cancer Soc.

[25] Lidgren A, Bergh A, Grankvist K, Rasmuson T, Ljungberg B (2008). Glucose transporter-1 expression in renal cell carcinoma and its correlation with hypoxia inducible factor-1 alpha. Bju Int.

[26] Lopez-Serra P, Marcilla M, Villanueva A, Ramos-Fernandez A, Palau A, Leal L (2016). Corrigendum: A DERL3-associated defect in the degradation of SLC2A1 mediates the Warburg effect. Nat Commun.

[27] Lopez-Serra P, Marcilla M, Villanueva A, Ramos-Fernandez A, Palau A, Leal L (2014). A DERL3-associated defect in the degradation of SLC2A1 mediates the Warburg effect. Nat Commun.

[28] Parton RG, Simons K (2007). The multiple faces of caveolae. Nat Rev Mol Cell Biol.

[29] Ha TK, Her NG, Lee MG, Ryu BK, Lee JH, Han J (2012). Caveolin-1 increases aerobic glycolysis in colorectal cancers by stimulating HMGA1-mediated GLUT3 transcription. Cancer Res.

[30] Brown NJ, Higham SE, Perunovic B, Arafa M, Balasubramanian S, Rehman I (2013). Lactate dehydrogenase-B is silenced by promoter methylation in a high frequency of human breast cancers. Plos One.

[31] Ristic B, Bhutia YD, Ganapathy V (2017). Cell-surface G-protein-coupled receptors for tumor-associated metabolites: A direct link to mitochondrial dysfunction in cancer. Biochim Biophys Acta Rev Cancer.

[32] Chesnelong C, Chaumeil MM, Blough MD, Al-Najjar M, Stechishin OD, Chan JA (2014). Lactate dehydrogenase A silencing in IDH mutant gliomas. Neuro Oncol.

[33] Brown NJ, Higham SE, Perunovic B, Arafa M, Balasubramanian S, Rehman I (2013). Lactate dehydrogenase-B is silenced by promoter methylation in a high frequency of human breast cancers. Plos One.

[34] Anastasiou D, Yu Y, Israelsen WJ, Jiang JK, Boxer MB, Hong BS (2012). Pyruvate kinase M2 activators promote tetramer formation and suppress tumorigenesis. Nat Chem Biol.

[35] Noguchi T, Inoue H, Tanaka T (1986). The M1- and M2-type isozymes of rat pyruvate kinase are produced from the same gene by alternative RNA splicing. J Biol Chem.

[36] Singh S, Narayanan SP, Biswas K, Gupta A, Ahuja N, Yadav S (2017). Intragenic DNA methylation and BORIS-mediated cancer-specific splicing contribute to the Warburg effect. Proc Natl Acad Sci U S A.

[37] Singh S, Narayanan SP, Biswas K, Gupta A, Ahuja N, Yadav S (2017). Intragenic DNA methylation and BORIS-mediated cancer-specific splicing contribute to the Warburg effect. Proc Natl Acad Sci U S A.

[38] Kottakis F, Nicolay BN, Roumane A, Karnik R, Gu H, Nagle JM (2016). LKB1 loss links serine metabolism to DNA methylation and tumorigenesis. Nature.

[39] DeWaal D, Nogueira V, Terry AR, Patra KC, Jeon SM, Guzman G (2018). Author Correction: Hexokinase-2 depletion inhibits glycolysis and induces oxidative phosphorylation in hepatocellular carcinoma and sensitizes to metformin. Nat Commun.

[40] Patra KC, Wang Q, Bhaskar PT, Miller L, Wang Z, Wheaton W (2013). Hexokinase 2 is required for tumor initiation and maintenance and its systemic deletion is therapeutic in mouse models of cancer. Cancer Cell.

[41] Thakur C, Chen F (2019). Connections between metabolism and epigenetics in cancers. Semin Cancer Biol.

[42] Wolf A, Agnihotri S, Munoz D, Guha A (2011). Developmental profile and regulation of the glycolytic enzyme hexokinase 2 in normal brain and glioblastoma multiforme. Neurobiol Dis.

[43] Lu J, Wang L, Chen W, Wang Y, Zhen S, Chen H (2019). miR-603 targeted hexokinase-2 to inhibit the malignancy of ovarian cancer cells. Arch Biochem Biophys.

[44] Zhang S, Pei M, Li Z, Li H, Liu Y, Li J (2018). Double-negative feedback interaction between DNA methyltransferase 3A and microRNA-145 in the Warburg effect of ovarian cancer cells. Cancer Sci.

[45] Zhong XY, Yuan XM, Xu YY, Yin M, Yan WW, Zou SW (2018). CARM1 Methylates GAPDH to Regulate Glucose Metabolism and Is Suppressed in Liver Cancer. Cell Rep.

[46] Polyak K, Li Y, Zhu H, Lengauer C, Willson JK, Markowitz SD (1998). Somatic mutations of the mitochondrial genome in human colorectal tumours. Nat Genet.

[47] Ohta S (2006). Contribution of somatic mutations in the mitochondrial genome to the development of cancer and tolerance against anticancer drugs. Oncogene.

[48] Gaowa S, Futamura M, Tsuneki M, Kamino H, Tajima JY, Mori R (2018). Possible role of p53/Mieap-regulated mitochondrial quality control as a tumor suppressor in human breast cancer. Cancer Sci.

[49] Tsuneki M, Nakamura Y, Kinjo T, Nakanishi R, Arakawa H (2015). Mieap suppresses murine intestinal tumor via its mitochondrial quality control. Sci Rep.

[50] Miyamoto Y, Kitamura N, Nakamura Y, Futamura M, Miyamoto T, Yoshida M (2011). Possible existence of lysosome-like organella within mitochondria and its role in mitochondrial quality control. Plos One.

[51] Iacobazzi V, Castegna A, Infantino V, Andria G (2013). Mitochondrial DNA methylation as a next-generation biomarker and diagnostic tool. Mol Genet Metab.

[52] Pollack Y, Kasir J, Shemer R, Metzger S, Szyf M (1984). Methylation pattern of mouse mitochondrial DNA. Nucleic Acids Res.

[53] Shmookler RR, Goldstein S (1983). Mitochondrial DNA in mortal and immortal human cells. Genome number, integrity, and methylation. J Biol Chem.

[54] Shock LS, Thakkar PV, Peterson EJ, Moran RG, Taylor SM (2011). DNA methyltransferase 1, cytosine methylation, and cytosine hydroxymethylation in mammalian mitochondria. Proc Natl Acad Sci U S A.

[55] Peterson EJ, Bogler O, Taylor SM (2003). p53-mediated repression of DNA methyltransferase 1 expression by specific DNA binding. Cancer Res.

[56] Dawid IB (1974). 5-methylcytidylic acid: absence from mitochondrial DNA of frogs and HeLa cells. Science.

[57] Nass MM (1973). Differential methylation of mitochondrial and nuclear DNA in cultured mouse, hamster and virus-transformed hamster cells. In vivo and in vitro methylation. J Mol Biol.

[58] Maekawa M, Taniguchi T, Higashi H, Sugimura H, Sugano K, Kanno T (2004). Methylation of mitochondrial DNA is not a useful marker for cancer detection. Clin Chem.

[59] Hong EE, Okitsu CY, Smith AD, Hsieh CL (2013). Regionally specific and genome-wide analyses conclusively demonstrate the absence of CpG methylation in human mitochondrial DNA. Mol Cell Biol.

[60] Ghosh S, Sengupta S, Scaria V (2014). Comparative analysis of human mitochondrial methylomes shows distinct patterns of epigenetic regulation in mitochondria. Mitochondrion.

[61] Shock LS, Thakkar PV, Peterson EJ, Moran RG, Taylor SM (2011). DNA methyltransferase 1, cytosine methylation, and cytosine hydroxymethylation in mammalian mitochondria. Proc Natl Acad Sci U S A.

[62] Saini SK, Mangalhara KC, Prakasam G, Bamezai R (2017). DNA Methyltransferase1 (DNMT1) Isoform3 methylates mitochondrial genome and modulates its biology. Sci Rep.

[63] van der Wijst MG, van Tilburg AY, Ruiters MH, Rots MG (2017). Experimental mitochondria-targeted DNA methylation identifies GpC methylation, not CpG methylation, as potential regulator of mitochondrial gene expression. Sci Rep.

[64] Varela P, Mastroianni KG, Ferrer H, Aranda C, Wallbach K, Ferreira DMG (2019). Functional Characterization and Pharmacological Evaluation of a Novel GLA Missense Mutation Found in a Severely Affected Fabry Disease Family. Nephron.

[65] Chai RC, Chang YZ, Wang QW, Zhang KN, Li JJ, Huang H (2019). A Novel DNA Methylation-Based Signature Can Predict the Responses of MGMT Promoter Unmethylated Glioblastomas to Temozolomide. Front Genet.

[66] Anderson S, Bankier AT, Barrell BG, de Bruijn MH, Coulson AR, Drouin J (1981). Sequence and organization of the human mitochondrial genome. Nature.

[67] Infantino V, Castegna A, Iacobazzi F, Spera I, Scala I, Andria G (2011). Impairment of methyl cycle affects mitochondrial methyl availability and glutathione level in Down's syndrome. Mol Genet Metab.

[68] Palmieri F (2013). The mitochondrial transporter family SLC25: identification, properties and physiopathology. Mol Aspects Med.

[69] Menga A, Palmieri EM, Cianciulli A, Infantino V, Mazzone M, Scilimati A (2017). SLC25A26 overexpression impairs cell function via mtDNA hypermethylation and rewiring of methyl metabolism. Febs J.

[70] Nakamura Y, Arakawa H (2017). Discovery of Mieap-regulated mitochondrial quality control as a new function of tumor suppressor p53. Cancer Sci.

[71] Tsuneki M, Nakamura Y, Kinjo T, Nakanishi R, Arakawa H (2015). Mieap suppresses murine intestinal tumor via its mitochondrial quality control. Sci Rep.

[72] Ciechanover A (2005). Proteolysis: from the lysosome to ubiquitin and the proteasome. Nat Rev Mol Cell Biol.

[73] Dice JF (2007). Chaperone-mediated autophagy. Autophagy.

[74] Miyamoto Y, Kitamura N, Nakamura Y, Futamura M, Miyamoto T, Yoshida M (2011). Possible existence of lysosome-like organella within mitochondria and its role in mitochondrial quality control. Plos One.

[75] Bonnet S, Archer SL, Allalunis-Turner J, Haromy A, Beaulieu C, Thompson R (2007). A mitochondria-K+ channel axis is suppressed in cancer and its normalization promotes apoptosis and inhibits cancer growth. Cancer Cell.

[76] Plas DR, Thompson CB (2002). Cell metabolism in the regulation of programmed cell death. Trends Endocrinol Metab.

[77] Leclerc D, Levesque N, Cao Y, Deng L, Wu Q, Powell J (2013). Genes with aberrant expression in murine preneoplastic intestine show epigenetic and expression changes in normal mucosa of colon cancer patients. Cancer Prev Res (Phila).

[78] Selak MA, Armour SM, MacKenzie ED, Boulahbel H, Watson DG, Mansfield KD (2005). Succinate links TCA cycle dysfunction to oncogenesis by inhibiting HIF-alpha prolyl hydroxylase. Cancer Cell.

[79] Isaacs JS, Jung YJ, Mole DR, Lee S, Torres-Cabala C, Chung YL (2005). HIF overexpression correlates with biallelic loss of fumarate hydratase in renal cancer: novel role of fumarate in regulation of HIF stability. Cancer Cell.

[80] Schofield CJ, Ratcliffe PJ (2004). Oxygen sensing by HIF hydroxylases. Nat Rev Mol Cell Biol.

[81] Vander HM, Cantley LC, Thompson CB (2009). Understanding the Warburg effect: the metabolic requirements of cell proliferation. Science.

[82] Liu H, Huang D, McArthur DL, Boros LG, Nissen N, Heaney AP (2010). Fructose induces transketolase flux to promote pancreatic cancer growth. Cancer Res.

[83] Jayachandran A, Lo PH, Chueh AC, Prithviraj P, Molania R, Davalos-Salas M (2016). Transketolase-like 1 ectopic expression is associated with DNA hypomethylation and induces the Warburg effect in melanoma cells. Bmc Cancer.

[84] Sun W, Liu Y, Glazer CA, Shao C, Bhan S, Demokan S (2010). TKTL1 is activated by promoter hypomethylation and contributes to head and neck squamous cell carcinoma carcinogenesis through increased aerobic glycolysis and HIF1alpha stabilization. Clin Cancer Res.

[85] Itoh K, Chiba T, Takahashi S, Ishii T, Igarashi K, Katoh Y (1997). An Nrf2/small Maf heterodimer mediates the induction of phase II detoxifying enzyme genes through antioxidant response elements. Biochem Biophys Res Commun.

[86] Uruno A, Motohashi H (2011). The Keap1-Nrf2 system as an in vivo sensor for electrophiles. Nitric Oxide.

[87] Shibata T, Ohta T, Tong KI, Kokubu A, Odogawa R, Tsuta K (2008). Cancer related mutations in NRF2 impair its recognition by Keap1-Cul3 E3 ligase and promote malignancy. Proc Natl Acad Sci U S A.

[88] Solis LM, Behrens C, Dong W, Suraokar M, Ozburn NC, Moran CA (2010). Nrf2 and Keap1 abnormalities in non-small cell lung carcinoma and association with clinicopathologic features. Clin Cancer Res.

[89] Mitsuishi Y, Taguchi K, Kawatani Y, Shibata T, Nukiwa T, Aburatani H (2012). Nrf2 redirects glucose and glutamine into anabolic pathways in metabolic reprogramming. Cancer Cell.

[90] Taguchi K, Motohashi H, Yamamoto M (2011). Molecular mechanisms of the Keap1-Nrf2 pathway in stress response and cancer evolution. Genes Cells.

[91] Sekhar KR, Yan XX, Freeman ML (2002). Nrf2 degradation by the ubiquitin proteasome pathway is inhibited by KIAA0132, the human homolog to INrf2. Oncogene.

[92] McMahon M, Thomas N, Itoh K, Yamamoto M, Hayes JD (2004). Redox-regulated turnover of Nrf2 is determined by at least two separate protein domains, the redox-sensitive Neh2 degron and the redox-insensitive Neh6 degron. J Biol Chem.

[93] Muscarella LA, Barbano R, D'Angelo V, Copetti M, Coco M, Balsamo T (2011). Regulation of KEAP1 expression by promoter methylation in malignant gliomas and association with patient's outcome. Epigenetics-Us.

[94] Muscarella LA, Parrella P, D'Alessandro V, la Torre A, Barbano R, Fontana A (2011). Frequent epigenetics inactivation of KEAP1 gene in non-small cell lung cancer. Epigenetics-Us.

[95] Cai D, Jia Y, Song H, Sui S, Lu J, Jiang Z (2014). Betaine supplementation in maternal diet modulates the epigenetic regulation of hepatic gluconeogenic genes in neonatal piglets. Plos One.

[96] Dong Y, Huaying S, Danying W, Chihong Z, Ruibin J, Xiaojiang S (2018). Significance of Methylation of FBP1 Gene in Non-Small Cell Lung Cancer. Biomed Res Int.

[97] Hirata H, Sugimachi K, Komatsu H, Ueda M, Masuda T, Uchi R (2016). Decreased Expression of Fructose-1,6-bisphosphatase Associates with Glucose Metabolism and Tumor Progression in Hepatocellular Carcinoma. Cancer Res.

[98] Dong C, Yuan T, Wu Y, Wang Y, Fan TW, Miriyala S (2013). Loss of FBP1 by Snail-mediated repression provides metabolic advantages in basal-like breast cancer. Cancer Cell.

[99] Liu X, Wang X, Zhang J, Lam EK, Shin VY, Cheng AS (2010). Warburg effect revisited: an epigenetic link between glycolysis and gastric carcinogenesis. Oncogene.

[100] Karpathakis A, Dibra H, Pipinikas C, Feber A, Morris T, Francis J (2016). Prognostic Impact of Novel Molecular Subtypes of Small Intestinal Neuroendocrine Tumor. Clin Cancer Res.

[101] Chen M, Zhang J, Li N, Qian Z, Zhu M, Li Q (2011). Promoter hypermethylation mediated downregulation of FBP1 in human hepatocellular carcinoma and colon cancer. Plos One.

[102] Li H, Wang J, Xu H, Xing R, Pan Y, Li W (2013). Decreased fructose-1,6-bisphosphatase-2 expression promotes glycolysis and growth in gastric cancer cells. Mol Cancer.

[103] Li B, Qiu B, Lee DS, Walton ZE, Ochocki JD, Mathew LK (2014). Fructose-1,6-bisphosphatase opposes renal carcinoma progression. Nature.

[104] Christofk HR, Vander HM, Harris MH, Ramanathan A, Gerszten RE, Wei R (2008). The M2 splice isoform of pyruvate kinase is important for cancer metabolism and tumour growth. Nature.

[105] Yang W, Lu Z (2013). Regulation and function of pyruvate kinase M2 in cancer. Cancer Lett.

[106] Mazurek S, Boschek CB, Hugo F, Eigenbrodt E (2005). Pyruvate kinase type M2 and its role in tumor growth and spreading. Semin Cancer Biol.

[107] Koeck T, Olsson AH, Nitert MD, Sharoyko VV, Ladenvall C, Kotova O (2011). A common variant in TFB1M is associated with reduced insulin secretion and increased future risk of type 2 diabetes. Cell Metab.

[108] Li B, Qiu B, Lee DS, Walton ZE, Ochocki JD, Mathew LK (2014). Fructose-1,6-bisphosphatase opposes renal carcinoma progression. Nature.

[109] Marin-Hernandez A, Rodriguez-Enriquez S, Vital-Gonzalez PA, Flores-Rodriguez FL, Macias-Silva M, Sosa-Garrocho M (2006). Determining and understanding the control of glycolysis in fast-growth tumor cells. Flux control by an over-expressed but strongly product-inhibited hexokinase. Febs J.

[110] Jitrapakdee S (2012). Transcription factors and coactivators controlling nutrient and hormonal regulation of hepatic gluconeogenesis. Int J Biochem Cell Biol.

[111] Yoon JC, Puigserver P, Chen G, Donovan J, Wu Z, Rhee J (2001). Control of hepatic gluconeogenesis through the transcriptional coactivator PGC-1. Nature.

[112] Lachance G, Uniacke J, Audas TE, Holterman CE, Franovic A, Payette J (2014). DNMT3a epigenetic program regulates the HIF-2alpha oxygen-sensing pathway and the cellular response to hypoxia. Proc Natl Acad Sci U S A.

[113] Li L, Liang Y, Kang L, Liu Y, Gao S, Chen S (2018). Transcriptional Regulation of the Warburg Effect in Cancer by SIX1. Cancer Cell.

[114] Epstein AC, Gleadle JM, McNeill LA, Hewitson KS, O'Rourke J, Mole DR (2001). C. elegans EGL-9 and mammalian homologs define a family of dioxygenases that regulate HIF by prolyl hydroxylation. Cell.

[115] Bruick RK, McKnight SL (2001). A conserved family of prolyl-4-hydroxylases that modify HIF. Science.

[116] Choudhry H, Harris AL (2018). Advances in Hypoxia-Inducible Factor Biology. Cell Metab.

[117] Ivan M, Kondo K, Yang H, Kim W, Valiando J, Ohh M (2001). HIFalpha targeted for VHL-mediated destruction by proline hydroxylation: implications for O2 sensing. Science.

[118] Jaakkola P, Mole DR, Tian YM, Wilson MI, Gielbert J, Gaskell SJ (2001). Targeting of HIF-alpha to the von Hippel-Lindau ubiquitylation complex by O2-regulated prolyl hydroxylation. Science.

[119] Maxwell PH, Wiesener MS, Chang GW, Clifford SC, Vaux EC, Cockman ME (1999). The tumour suppressor protein VHL targets hypoxia-inducible factors for oxygen-dependent proteolysis. Nature.

[120] Kaelin WJ, Ratcliffe PJ (2008). Oxygen sensing by metazoans: the central role of the HIF hydroxylase pathway. Mol Cell.

[121] Wenger RH, Stiehl DP, Camenisch G (2005). Integration of oxygen signaling at the consensus HRE. Sci STKE.

[122] Kattygnarath D, Maugenre S, Neyroud N, Balse E, Ichai C, Denjoy I (2011). MOG1: a new susceptibility gene for Brugada syndrome. Circ Cardiovasc Genet.

[123] Vaupel P, Fortmeyer HP, Runkel S, Kallinowski F (1987). Blood flow, oxygen consumption, and tissue oxygenation of human breast cancer xenografts in nude rats. Cancer Res.

[124] Schodel J, Grampp S, Maher ER, Moch H, Ratcliffe PJ, Russo P (2016). Hypoxia, Hypoxia-inducible Transcription Factors, and Renal Cancer. Eur Urol.

[125] Ginsburg KS, Weber CR, Bers DM (2013). Cardiac Na+-Ca2+ exchanger: dynamics of Ca2+-dependent activation and deactivation in intact myocytes. J Physiol.

[126] Minchenko O, Opentanova I, Caro J (2003). Hypoxic regulation of the 6-phosphofructo-2-kinase/fructose-2,6-bisphosphatase gene family (PFKFB-1-4) expression in vivo. Febs Lett.

[127] Minchenko OH, Ogura T, Opentanova IL, Minchenko DO, Esumi H (2005). Splice isoform of 6-phosphofructo-2-kinase/fructose-2,6-bisphosphatase-4: expression and hypoxic regulation. Mol Cell Biochem.

[128] Keith B, Johnson RS, Simon MC (2011). HIF1alpha and HIF2alpha: sibling rivalry in hypoxic tumour growth and progression. Nat Rev Cancer.

[129] Dabral S, Muecke C, Valasarajan C, Schmoranzer M, Wietelmann A, Semenza GL (2019). A RASSF1A-HIF1alpha loop drives Warburg effect in cancer and pulmonary hypertension. Nat Commun.

[130] Kim JW, Tchernyshyov I, Semenza GL, Dang CV (2006). HIF-1-mediated expression of pyruvate dehydrogenase kinase: a metabolic switch required for cellular adaptation to hypoxia. Cell Metab.

[131] Koslowski M, Luxemburger U, Tureci O, Sahin U (2011). Tumor-associated CpG demethylation augments hypoxia-induced effects by positive autoregulation of HIF-1alpha. Oncogene.

[132] Fox SB, Braganca J, Turley H, Campo L, Han C, Gatter KC (2004). CITED4 inhibits hypoxia-activated transcription in cancer cells, and its cytoplasmic location in breast cancer is associated with elevated expression of tumor cell hypoxia-inducible factor 1alpha. Cancer Res.

[133] Arany Z, Huang LE, Eckner R, Bhattacharya S, Jiang C, Goldberg MA (1996). An essential role for p300/CBP in the cellular response to hypoxia. Proc Natl Acad Sci U S A.

[134] Huang KT, Takano EA, Mikeska T, Byrne DJ, Dobrovic A, Fox SB (2011). Aberrant DNA methylation but not mutation of CITED4 is associated with alteration of HIF-regulated genes in breast cancer. Breast Cancer Res Treat.

[135] O'Keefe LV, Colella A, Dayan S, Chen Q, Choo A, Jacob R (2011). Drosophila orthologue of WWOX, the chromosomal fragile site FRA16D tumour suppressor gene, functions in aerobic metabolism and regulates reactive oxygen species. Hum Mol Genet.

[136] Abu-Remaileh M, Aqeilan RI (2014). Tumor suppressor WWOX regulates glucose metabolism via HIF1alpha modulation. Cell Death Differ.

[137] Abu-Remaileh M, Aqeilan RI (2015). The tumor suppressor WW domain-containing oxidoreductase modulates cell metabolism. Exp Biol Med (Maywood).

[138] Ekizoglu S, Bulut P, Karaman E, Kilic E, Buyru N (2015). Epigenetic and genetic alterations affect the WWOX gene in head and neck squamous cell carcinoma. Plos One.

[139] Foxler DE, Bridge KS, James V, Webb TM, Mee M, Wong SC (2012). The LIMD1 protein bridges an association between the prolyl hydroxylases and VHL to repress HIF-1 activity. Nat Cell Biol.

[140] Chakraborty C, Mitra S, Roychowdhury A, Samadder S, Dutta S, Roy A (2018). Deregulation of LIMD1-VHL-HIF-1alpha-VEGF pathway is associated with different stages of cervical cancer. Biochem J.

[141] Hu CJ, Wang LY, Chodosh LA, Keith B, Simon MC (2003). Differential roles of hypoxia-inducible factor 1alpha (HIF-1alpha) and HIF-2alpha in hypoxic gene regulation. Mol Cell Biol.

[142] Lachance G, Uniacke J, Audas TE, Holterman CE, Franovic A, Payette J (2014). DNMT3a epigenetic program regulates the HIF-2alpha oxygen-sensing pathway and the cellular response to hypoxia. Proc Natl Acad Sci U S A.

[143] Rawluszko-Wieczorek AA, Horbacka K, Krokowicz P, Misztal M, Jagodzinski PP (2014). Prognostic potential of DNA methylation and transcript levels of HIF1A and EPAS1 in colorectal cancer. Mol Cancer Res.

[144] Stover PJ (2004). Physiology of folate and vitamin B12 in health and disease. Nutr Rev.

[145] Chen WJ, Huang RS (2018). Low-folate stress reprograms cancer stem cell-like potentials and bioenergetics metabolism through activation of mTOR signaling pathway to promote in vitro invasion and in vivo tumorigenicity of lung cancers. J Nutr Biochem.

[146] Wang TP, Hsu SH, Feng HC, Huang RF (2012). Folate deprivation enhances invasiveness of human colon cancer cells mediated by activation of sonic hedgehog signaling through promoter hypomethylation and cross action with transcription nuclear factor-kappa B pathway. Carcinogenesis.

[147] Fan J, Ye J, Kamphorst JJ, Shlomi T, Thompson CB, Rabinowitz JD (2014). Quantitative flux analysis reveals folate-dependent NADPH production. Nature.

[148] Ding K, Jiang J, Chen L, Xu X (2018). Methylenetetrahydrofolate Dehydrogenase 1 Silencing Expedites the Apoptosis of Non-Small Cell Lung Cancer Cells via Modulating DNA Methylation. Med Sci Monit.

[149] Nilsson R, Jain M, Madhusudhan N, Sheppard NG, Strittmatter L, Kampf C (2014). Metabolic enzyme expression highlights a key role for MTHFD2 and the mitochondrial folate pathway in cancer. Nat Commun.

[150] Zheng LD, Linarelli LE, Liu L, Wall SS, Greenawald MH, Seidel RW (2015). Insulin resistance is associated with epigenetic and genetic regulation of mitochondrial DNA in obese humans. Clin Epigenetics.

[151] Mentch SJ, Mehrmohamadi M, Huang L, Liu X, Gupta D, Mattocks D (2015). Histone Methylation Dynamics and Gene Regulation Occur through the Sensing of One-Carbon Metabolism. Cell Metab.

[152] Maddocks OD, Labuschagne CF, Adams PD, Vousden KH (2016). Serine Metabolism Supports the Methionine Cycle and DNA/RNA Methylation through De Novo ATP Synthesis in Cancer Cells. Mol Cell.

[153] Locasale JW (2013). Serine, glycine and one-carbon units: cancer metabolism in full circle. Nat Rev Cancer.

[154] Chang CH, Curtis JD, Maggi LJ, Faubert B, Villarino AV, O'Sullivan D (2013). Posttranscriptional control of T cell effector function by aerobic glycolysis. Cell.

[155] Peng M, Yin N, Chhangawala S, Xu K, Leslie CS, Li MO (2016). Aerobic glycolysis promotes T helper 1 cell differentiation through an epigenetic mechanism. Science.

[156] Kramer AC, Kothari A, Wilson WC, Celik H, Nikitas J, Mallaney C (2017). Dnmt3a regulates T-cell development and suppresses T-ALL transformation. Leukemia.

[157] Zhang D, Li J, Wang F, Hu J, Wang S, Sun Y (2014). 2-Deoxy-D-glucose targeting of glucose metabolism in cancer cells as a potential therapy. Cancer Lett.

[158] Zhou L, Liu L, Chai W, Zhao T, Jin X, Guo X (2019). Dichloroacetic acid upregulates apoptosis of ovarian cancer cells by regulating mitochondrial function. Onco Targets Ther.

[159] Mathupala SP, Ko YH, Pedersen PL (2009). Hexokinase-2 bound to mitochondria: cancer's stygian link to the "Warburg Effect" and a pivotal target for effective therapy. Semin Cancer Biol.

[160] Idelchik M, Begley U, Begley TJ, Melendez JA (2017). Mitochondrial ROS control of cancer. Semin Cancer Biol.

[161] Christman JK (2002). 5-Azacytidine and 5-aza-2'-deoxycytidine as inhibitors of DNA methylation: mechanistic studies and their implications for cancer therapy. Oncogene.

[162] Pulliam N, Fang F, Ozes AR, Tang J, Adewuyi A, Keer H (2018). An Effective Epigenetic-PARP Inhibitor Combination Therapy for Breast and Ovarian Cancers Independent of BRCA Mutations. Clin Cancer Res.

[163] Zhou S, Li Y, Huang F, Zhang B, Yi T, Li Z (2012). Live-attenuated measles virus vaccine confers cell contact loss and apoptosis of ovarian cancer cells via ROS-induced silencing of E-cadherin by methylation. Cancer Lett.

[164] Das DS, Ray A, Das A, Song Y, Tian Z, Oronsky B (2016). A novel hypoxia-selective epigenetic agent RRx-001 triggers apoptosis and overcomes drug resistance in multiple myeloma cells. Leukemia.

